# The Impact of Devolution on Local Health System Financing: A Synthetic Difference-in-Differences Study of Greater Manchester, England

**DOI:** 10.34172/ijhpm.8689

**Published:** 2025-09-20

**Authors:** Charlie Moss, Philip Britteon, Yiu-Shing Lau, Laura Anselmi

**Affiliations:** Health Organisation, Policy and Economics (HOPE), Centre for Primary Care and Health Services Research, The University of Manchester, Manchester, UK.

**Keywords:** Devolution, Decentralization, Health Expenditure, Local Government Expenditure, Health Policy, United Kingdom

## Abstract

**Background:**

Determining the effects of devolution policies through health system financing is pivotal in understanding their impact. Few existing studies have considered total health and care system expenditure, overlooking the transfer of resources through spending in different services locally. We evaluated the impact of devolution in Greater Manchester (GM), an area in England which received devolved health and social care powers from 2016, on the whole system of local public expenditures on health and care.

**Methods:**

Using data on public health and care spending for 149 local health systems between 2013 and 2020, we estimated synthetic difference-in-differences (DiD). We compared expenditure in total and by services for ten GM localities relative to a weighted combination of localities from the rest of England (excluding London) for four years post-devolution. We analysed expenditures in per capita terms and as a share of total expenditure. We investigated dynamic effects with an event study specification.

**Results:**

Compared with the synthetic control (SC) group, total annual expenditure on health and care increased in GM post-devolution by an average of £66.58 per-capita (95% CI: 11.85 to 121.30). Total expenditure on public health and social care, managed by Local Authorities, increased by £36.39 (95% CI: 6.99 to 65.80) and expenditure on social care specifically increased in the third and fourth years after devolution. We detected some short-term changes in expenditure managed by the National Health Service (NHS) Clinical Commissioning Groups (CCGs), including reduced expenditure on Continuing Healthcare and increased expenditure on acute healthcare and "other" miscellaneous expenditure. We did not detect a statistically significant effect for public health, primary care, community or mental healthcare.

**Conclusion:**

Results suggest that additional resources were used to respond to existing pressures on the health system, and that to redirect expenditure substantial increase in resources or re-organisation of services may be required alongside devolution of sufficient powers.

## Introduction

**Key Messages**

**Implications for policy makers**

- Devolution was associated with increased spending in overall health and social care in Greater Manchester (GM), likely financed mainly by transformation funding received from the UK government as part of the devolution deal.
- There were limited changes in spending on National Health Service (NHS) health services, except for a short-term increase in acute care, “other” miscellaneous expenditure, and a short-term reduction in spending on Continuing Healthcare. Increased autonomy and resources were not sufficient to change spending patterns and shift resources towards preventive services in line with GM strategic objectives.
- Additional resources and sufficient powers to enable a substantial service re-organisation may be required for local health systems to change patterns of spending.

**Implications for the public**

Decentralisation is a common policy in the health and care sector, whereby central governments transfer some responsibilities for those services to lower levels of government to enable them to be more responsive to specific population needs. We analysed whether devolution of health and social care in Greater Manchester (GM) changed how much local organisations spent on health and care, or which services they spent on. We found that health and care spending increased after devolution, mainly funded by transformation funding that was part of the devolution deal. There were short-term increases in the National Health Service (NHS) spending on acute hospital services, and “other” miscellaneous healthcare expenditure. There were increases in social care spending managed by Local Authorities several years after devolution. However, changes were not in line with GM’s strategic policy objective of increasing spending on many of the (more preventive) services. This suggests that some devolved powers and some additional funding may not be sufficient for changing patterns of health and care expenditure. Increased resources and sufficient powers to enable a substantial service re-organisation may be required.

Many countries have implemented devolution and decentralisation policies to improve the efficiency and/or equity of public services, particularly in the health and care sector.^1^ This push towards transferring authority and resources has been driven by the notion that local decision-makers are more aligned with and able to address the needs of their population.^2,3^ However, the potential benefits could also lead to disparities in care, reduce co-ordination between regions, and forego the economies of scale benefitted by more centralised systems.^4^

 An “ideal” health system likely integrates both centralised and decentralised elements, enabling local accountability while exploiting economies of scale and mitigating inter-regional inequalities. Determining the optimal balance between these elements for improved health system performance has been the focus of extensive research, however findings are often context-specific and not universally generalisable.^5-9^ In practice, devolution policies are highly varied, shaped by institutional context, political dynamics, and human agency.^9^

 Nonetheless, one broadly supported conclusion from the literature is that financing is a pivotal mechanism shaping the overall health system impact of devolution.^1^ Some studies have focused on financing arrangements built into the devolution policy or related mechanisms that remain centrally determined, such as regional cross-subsidisation.^6^ Other studies have investigated the financing decisions made by sub-national authorities following devolution or decentralisation, often focussing on the total resources allocated to healthcare, either in absolute terms or as a proportion of overall budget.^10-13^

 An equally important question is how devolution impacts the distribution of resources across different programs of local health and care expenditure. Quantitative empirical evidence is extremely limited. One study of decentralisation in Indonesia found that while resources allocated to health increased significantly following decentralisation, the increase was not evenly distributed over different lines of health spending.^14^ Spending by districts increased on some budget lines with more immediately tangible benefits (such as direct health services and equipment), whereas it remained unchanged on budget lines with less immediately tangible benefits (health promotion, monitoring and evaluation, and public-goods-related activities).^14^ Local spending priorities may shift following decentralisation either because local decision-makers are responding to local needs, or because the incentive structures they face differ from those shaping decisions at the national level. By examining the composition of expenditure rather than focusing solely on total spending, it is possible to gain deeper insight into the mechanisms that shape positive and negative impacts of devolution policies.

 We evaluated the impact of health and social care devolution on financial flows across the whole health and social care system in Greater Manchester (GM), a region in the North-West of England. In April 2016, responsibilities for the implementation of health and care services were devolved from the UK government to GM, following a set of wider devolution deals over other public services.^15,16^ This devolution deal allowed local commissioners of health and care services greater flexibility in how they spent and allocated resources, and gave the region access to £450m of additional funding for the purpose of service transformation.^17^ Unlike past devolution deals in other countries, however, fiscal powers and resources remained relatively centralised and GM was still subject to the same national targets, core budget allocations, and rules as elsewhere in the country.^18^ It is for these reasons that the GM devolution deal has been described as a “soft” form of devolution.^18^

 Within England, GM devolution provides a useful case study for other areas considering health devolution agreements. GM was the first region in England to agree devolved powers over budgets for health and care and is still the most extensive.^19^ More generally, GM provides a useful case study of “soft” devolution and has specific policy relevance for other countries with stable health and care resources that are centrally pooled and primarily redistributed to local levels through government transfers.

 A recent evaluation of GM devolution identified improvements in primary care, emergency, and inpatient services; cancer screening rates; social determinants of health; and life expectancy relative to comparable areas from the rest of England.^17,20^ The evaluation also investigated changes in resource availability, including spending, staffing, and beds, but overlooked the impact of devolution on financial flows and overall spending within the health system.

 We considered how devolution affected financing across the whole health system, building on a separate descriptive study describing the flow of health and social care funding in local health systems, with an illustration for GM over the period following the devolution deal.^21^ Using a synthetic control (SC) group from the rest of England, we examined the impact on health and social care expenditure in total; expenditure on healthcare services managed by Clinical Commissioning Groups (CCGs) and on social care and public health services managed by Local Authorities; and the composition of total expenditure by healthcare service sector, social care and public health. Within a difference-in-differences (DiD) framework, we used an event study to examine dynamic effects on each expenditure outcome, in addition to the average post-devolution effect.

## Contextualising Devolution in Greater Manchester

###  Health and Care Financing in England

 Most health and social care provision in the United Kingdom is government-financed through taxation (83% of total healthcare and 69% of long-term care in 2021).^22^ Healthcare resources are pooled centrally and distributed to local organisations in charge of commissioning services to address the needs of their populations. The National Health Service (NHS) primary and secondary care services are provided locally. Primary medical care is provided by general practices, while secondary care is provided by a mixture of NHS trusts (hospitals), private sector, and third sector providers.^23^

 During the study period (2013/2014 to 2019/2020), general practitioner and nurse-led organisations, known as CCGs, were responsible for commissioning (ie, purchasing) core NHS healthcare services for their local populations.^24^ Services commissioned by CCGs absorbed around two thirds of the total NHS budget. CCGs merged and reduced in number from 211 in 2013/2014 to 191 in 2019/2020. During the study period, 152 Local Authorities (each led by a council of locally elected representatives) were responsible for commissioning social care and most public health services, such as sexual health, smoking cessation, and drugs and alcohol misuse treatment.

 In England a distinction has usually been made in commissioning between: Primary care (including general practice services, optometry, and dentistry), secondary care (including general and acute hospital services and mental health), community care (including community nursing and some other community-based services such as physiotherapy) and specialised care (low volume, high cost services).^25^ Needs-based formulae were used to allocate public resources to CCGs for secondary care services (the “core services” allocation), but funding was not earmarked for specific services.^24^ Payments to general practices for primary care services were made centrally until 2014, when CCGs were given the opportunity to take on a greater role either through “co-commissioning” or up to full delegation of the primary care budget. CCGs could apply for primary care commissioning responsibilities from 2015/2016 and adoption of those responsibilities was staggered between 2015/2016 and 2021/2022.^25^ Two GM CCGs adopted delegated co-commissioning of primary care in 2015/2016, and the remaining ten adopted it in 2016/2017. It is worth noting that over half of total payments to general practices were still determined by a centrally defined capitation formula.^26^ Specialised services remained commissioned centrally.^24^

 Local Authorities financed local services, including social care and public health, using a mixture of central government grants, council tax (on residential property), and business rates (tax on commercial property).^27^ The public health grant was one of the few ring-fenced central government grants. During the study period councils retained all council tax revenue, most Local Authorities retained 50% of business rates and paid the remainder to the central government. From 2017/2018, however, a subset of Local Authorities including those in GM participated in a 100% business rate retention pilot, permitting them to retain all real terms growth in business rate revenues.^28^

###  Devolution in Greater Manchester 

 GM is an area in the North-West of England with a population of around 2.8m. In 2009, GM was given City Region status and allowed to establish a combined authority with formal delegated powers for public transport, skills, housing, planning, and economic regeneration.^15^ In 2014, a devolution agreement set out further devolution of powers for planning, land, transport, and fire services, as part of a wider set of devolution deals with other regions in England.^19^ These powers have been expanded over time to additional services, both in GM and other regions in England. However, GM was the first region to agree a devolution deal and has been granted the most devolved powers to date, outside of London.^29^

 In July 2015, GM and NHS England also made a unique agreement to devolve health and social care in the region,^30^ after which a devolution transition team operated in shadow form until April 2016.^18,30^ When the health and social care devolution deal came into effect in April 2016, Greater Manchester Health and Social Care Strategic Partnership Board was formed, bringing together 37 GM organisations including 12 CCGs, 10 Local Authorities, and 15 NHS and foundation trusts.^16^ The organisations had a rich history of collaboration for several decades prior to devolution,^18^ and an ambition to shift resources towards more preventive services following devolution.^16,21^ GM was still subject to all targets and regulations prescribed by the central government.^18,31^

 Revenue-raising powers were not devolved.^32^ However, GM did receive £450m of transformation funding from the UK government as part of the devolution deal in April 2016, which was subsequently distributed across the ten localities at the discretion of the Partnership from July 2016 onwards to assist with strategic health system transformation.^18,33^ Other regions throughout the rest of England also received sustainability and transformation funding, but in those cases it remained controlled centrally by NHS England and was mainly used to plug provider deficits.^34^ Additionally, GM’s participation in the 100% business rates retention scheme pilot from 2017/2018 provided further opportunity for GM Local Authorities to increase their revenues, by increasing real terms growth in business rates revenues.^17,35^ The financial benefit of the scheme to pilot areas may have amounted to around a 3.6% increase in their core spending power.^35^

###  Conceptualisation

 A variety of conceptual frameworks have been used to describe and analyse health sector decentralisation.^36,37^ Bossert’s (1998) framework centres around the concept of “decision space,” defined as the freedom granted by central government to local organisations, and has previously been used to analyse health service commissioning in England.^38,39^ First, ‘decision space’ is defined through the functions (finance, service organisation, human resources, access rules, and governance rules) and degrees of choice formally transferred to local officials. Second, consideration is given to the choices made with the increased discretion, and to finally evaluate the impact of those choices on performance outcomes.

 We examined the choices made by local decision-makers in GM following devolution. We focused on decisions around spending in different health and care sectors, as defined in a previous study introducing a framework to describe the flow of funding in local systems, with an illustration for GM.^21^

 There are several ways in which devolution may have impacted health and care spending in GM. Additional resources from the transformation funding and the business rates retention scheme, alongside discretional powers and coordination mechanisms, effectively changed the decision space for local decision-makers.

## Data

 We used information on expenditure by financial year (1st of April to 31st of March of the following year) from 2013/2014 to 2019/2020 from three data sources.

 First, we used data on CCG expenditure gathered by NHS England. Based on CCG records, expenditure was classified into ten categories, including: Acute care, mental health, community, primary care, Continuing Healthcare (mainly nursing care arranged and funded by the NHS, for qualifying individuals with long-term complex care needs^40^), adult social care, administrative, corporate (such as chief executive officer/board office payments, financial accounting, strategy and development, corporate governance, travel costs), programme (such as business intelligence, digital/information technology expenditure), and other expenditure (such as expenditure on safeguarding, quality programmes, and service expenditure that is not easily placed into other specific categories). Amounts for adult social care were very small, with many annual expenditures taking the value of zero. We combined adult social care spending with Continuing Healthcare because they are services with similar objectives and for similar population groups. We also combined categories associated with CCGs’ running costs with miscellaneous expenditure (administrative, corporate, programme, and other). This yielded six categories of CCG expenditure: (*i*) Acute care; (*ii*) Mental health; (*iii*) Community health services; (*iv*) Primary care; (*v*) Continuing healthcare; and (*vi*) Other.

 Second, we used data from the NHS Payments to General Practice dataset to proxy primary care expenditure.^41^ We used “total NHS payments to general practice,” capturing both payments made centrally and by each CCG, instead of CCG reported expenditure, because this measure was not affected by the delegation of primary care commissioning responsibilities to CCGs at different times.

 Third, we used the revenue outturn for social care and public health services annual dataset published by the UK government, which collects expenditure of each Local Authority in England.^42^ We extracted information on three categories of Local Authority expenditure (net of any fees and charges) linked to the provision of health and care services: (*i*) Public health; (*ii*) Adult social care; (*iii*) Children’s social care.

 We mapped Local Authority expenditure to CCG geography using midyear population estimates from the Office for National Statistics (ONS) for each Lower Super Output Area (LSOA) in England. LSOAs are small geographic areas with an average population of around 1500 that map directly to Local Authority and CCG footprints. We calculated the weighted average Local Authority expenditure within each CCG from the proportion of the CCG population in each Local Authority. In instances where CCGs merged into a new unit during the study period, we created a “ghost unit” by calculating their population weighted average expenditure prior to the merger. This allowed us to track units over the whole study period while maximising the number of observations in the sample. The unit of analysis is therefore CCG geographies as they existed in 2019/2020 (N = 191).

 We used the gross domestic product deflator tool published by HM Treasury to convert all measures of expenditure to 2019/2020 prices. We then calculated per capita expenditure (using ONS midyear population estimates), share of total expenditure, and share of total CCG or Local Authority expenditure within each local health system. We also used ONS midyear estimates on the age-sex profile of the population at the LSOA level to derive information on the proportion of females and average age by sex of each CCG over time.

## Methods

 We estimated the impact of GM devolution on local health system expenditure using a combination of SC and DiD methods, to draw on the benefits of both as outlined by Arkhangelsky et al.^43^ DiD methods compare outcome changes over time between treated and control groups, under the assumption that they would have followed parallel trends in the absence of treatment.^44^ This allows DiD methods to account for unobserved, time-invariant differences between groups. Alternatively, SC methods relax the parallel trends assumption by constructing a weighted combination of control units (ie, SC) with pre-treatment outcome levels and trends similar to the treated group. This approach can be more robust but requires overlap in outcome levels to identify an appropriate comparator. By combining both approaches, we retain SC’s ability to account for differences in pre-treatment trends, while relaxing its requirement for close overlap in levels by controlling for time invariant differences in the DiD model.

 First, we used the SC method to derive weights for an SC group for each of the 10 CCGs in GM^45^ by minimising differences between each CCG in GM and the control group in: (*i*) demeaned per capita expenditure by service in each pre-devolution period (27 variables), and (*ii*) the age-sex composition of the population (3 variables). We assigned equal importance weights to each predictor variable to ensure comparability between the SC group for each CCG in GM across the different expenditure measures analysed. We limited the predictor set used to construct the SCs to historical expenditure patterns and age-sex composition in order to avoid overfitting the model used to derive the SC weights, in line with other studies.^17,20^

 Second, we estimated the impact of devolution on each measure of expenditure using a weighted DiD model:


$$y_{it}=\alpha +\beta GM_{i}+\delta \left(GM_{i}*Post_{t}\right)+\gamma_{t}+e_{it}$$

where *y*_it_ is the outcome for CCG i in year t, GM_i_ is a binary variable indicating if the CCG is in GM, Post_t_ is a binary variable indicating the post-devolution period (2016/2017 onwards), *γ*_t_are financial year fixed effects, and *e*_it_ is an error term. In this model, the coefficient δ estimated the average yearly impact of devolution on expenditure. We chose 2016/2017 as the first post-devolution year because the health and social care devolution deal was introduced in April 2016.

 We also estimated an event study specification, a generalisation of DiD that estimates the effect of an intervention at multiple time points, to examine annual changes in impact over time:


$$y_{it}=\alpha +\beta GM_{i}+\sum_{t=2016}^{2019}\delta_{t}\left(GM_{i}*year_{t}\right)+\gamma_{t}+e_{it}$$

This allowed us to explore how impacts evolved in the years before and after devolution occurred. We estimated each of these models using weighted least squares regression, with: (*i*) weights for each CCG in GM set equal to the share of the GM population to estimate the population weighted average impact; and (*ii*) weights for each control CCG set equal to their average weight across the 10 SC groups. The method estimated the causal impact of devolution on expenditure under the assumption that expenditure trends in GM would have followed the same expenditure trends as the weighted SC group throughout the post-devolution period, in the absence of the reform.

 In line with previous studies, we excluded CCGs in London from the pool of potential control units as the transformation of health and social care services towards greater devolution had already begun in London from 2015, with further progress made during the study period.^17,20,46^ This ensured that comparisons were made with CCGs where health and social care devolution had not already begun. After removing London CCGs, there were 149 potential control units from the rest of England.

 We also restricted the follow-up period to 2019/2020 to avoid attributing differences in expenditure to region-specific impacts of the COVID-19 pandemic. During the second half of March 2020 (the final two weeks of the 2019/2020 financial year) use of health services was affected by the COVID-19 pandemic in England.^47^ The first lockdown was initiated one week before the end of March. This is a small enough proportion of the year, and early enough in the pandemic, to allow the inclusion of 2019/2020.

 GM received additional powers and transformation funding in April 2016, and would have had limited mechanisms available to redistribute expenditure before that time. Anticipatory effects in the years prior to 2016/2017 are therefore unlikely, but it is still possible. We tested for parallel trends between GM and SC pre-devolution for each expenditure measure by estimating the joint significance of differences in expenditure in each period using an F-test, and the differences between linear trends in expenditure throughout the pre-devolution period.

###  Sensitivity Analysis

 We estimated a number of sensitivity analyses to investigate what could be driving the results.

 First, expenditure by GM CCGs could have differed from the rest of England for reasons unrelated to devolution, such as changes in the allocation of resources from the central government. To rule out this hypothesis, we investigated whether there was an effect of devolution on core allocations received by GM CCGs.

 Second, we estimated the impact of devolution on CCG primary care expenditure by restricting the SC group to CCGs that received delegated co-commissioning responsibilities at the same time as GM. This approach allowed us to exclude changes in expenditure driven by central commissioning, but relied on a smaller pool of control units.

 Third, we tested for potential spillover effects by excluding neighbouring CCGs from the pool of control units. Whilst the mechanisms for financial decision-making in the surrounding areas did not change, it is possible that patients may have sought care across regional borders driving changes in spending through demand. By excluding these CCGs we can determine the extent to which these changes, if any, affected our results.

## Results

###  Descriptive Statistics

 We first compared expenditure and population characteristics in GM with the SC.

 Estimated SC weights were non-negative for 65 of the 149 CCGs from the rest of England (Supplementary file 1, Table S1).

 Average population characteristics in the SC group were closer in magnitude to GM than the average in the rest of England (Table 1; Supplementary file 1, Table S2). However, the proportion of females, and mean age of the population were still higher in the SC group than in GM.

**Table 1:** Descriptive Statistics Before and After Devolution

	**GM**			**Rest of England**^a^			**SC**^b^		
	**Before Devolution** **(2013-2015)**	**After Devolution** **(2016-2019)**	**Difference**	**Before Devolution** **(2013-2015)**	**After Devolution** **(2016-2019)**	**Difference**	**Before Devolution** **(2013-2015)**	**After Devolution** **(2016-2019)**	**Difference**
Population characteristics									
Female (%)	50.47	50.33	-0.14	50.77	50.69	-0.08	50.55	50.44	-0.11
Female age (years)	39.40	39.57	0.17	41.97	42.34	0.37	40.28	40.57	0.30
Male age (years)	37.71	37.96	0.25	40.01	40.44	0.44	38.46	38.84	0.37
Per capita expenditures (£)									
Total	2009.50	2177.34	167.83	2087.90	2195.57	107.67	2313.18	2414.44	101.26
CCG Total	1373.96	1483.82	109.85	1422.10	1497.67	75.57	1566.78	1646.45	79.67
LA Total	635.54	693.52	57.98	665.80	697.90	32.10	746.40	767.99	21.59
CCG acute	801.20	852.41	51.21	803.66	835.92	32.26	885.75	918.44	32.69
CCG continuing healthcare	62.93	65.40	2.46	90.41	97.37	6.96	92.62	102.39	9.78
CCG community health services	141.89	150.45	8.56	142.87	149.77	6.90	147.91	152.55	4.64
CCG mental health	150.58	163.17	12.60	147.95	161.47	13.52	166.50	183.17	16.68
CCG other expenditure	73.21	90.66	17.45	62.07	68.75	6.68	85.66	87.13	1.47
Primary care	144.15	161.73	17.58	175.14	184.39	9.25	188.35	202.76	14.41
LA adult social care	384.39	411.11	26.73	432.10	445.24	13.14	462.54	470.46	7.92
LA children’s social care	175.34	197.48	22.14	174.33	187.84	13.50	210.03	218.01	7.99
LA public health	75.81	84.93	9.12	59.37	64.82	5.45	73.84	79.52	5.68
Share of total expenditure									
CCG Total	68.40	68.15	-0.25	68.14	68.26	0.12	67.73	68.10	0.37
LA Total	31.60	31.85	0.25	31.86	31.74	-0.12	32.27	31.90	-0.37
CCG acute	39.97	39.18	-0.78	38.51	38.10	-0.41	38.33	37.99	-0.34
CCG continuing healthcare	3.14	3.02	-0.12	4.34	4.45	0.11	4.03	4.25	0.22
CCG community health services	7.06	6.91	-0.15	6.83	6.83	-0.01	6.40	6.35	-0.04
CCG mental health	7.41	7.45	0.04	7.07	7.33	0.26	7.17	7.57	0.40
CCG other expenditure	3.64	4.15	0.51	2.96	3.11	0.16	3.67	3.57	-0.10
Primary care	7.18	7.43	0.25	8.43	8.44	0.01	8.13	8.37	0.23
LA adult social care	19.19	18.91	-0.28	20.77	20.31	-0.46	20.06	19.56	-0.50
LA children’s social care	8.67	9.04	0.37	8.29	8.51	0.23	9.02	9.04	0.02
LA public health	3.74	3.89	0.15	2.80	2.92	0.12	3.18	3.30	0.11
Share of CCG/LA expenditure									
CCG acute	58.42	57.50	-0.93	56.52	55.80	-0.72	56.57	55.78	-0.79
CCG continuing healthcare	4.60	4.44	-0.15	6.37	6.51	0.15	5.96	6.25	0.28
CCG community health services	10.32	10.14	-0.17	10.03	10.01	-0.02	9.45	9.35	-0.10
CCG mental health	10.86	10.93	0.07	10.39	10.76	0.37	10.62	11.14	0.52
CCG other expenditure	5.31	6.09	0.78	4.34	4.57	0.22	5.40	5.24	-0.16
Primary care	10.50	10.90	0.40	12.36	12.35	-0.01	11.99	12.24	0.25
LA adult social care	60.77	59.31	-1.46	65.32	64.09	-1.23	62.24	61.31	-0.92
LA children’s social care	27.38	28.43	1.06	25.91	26.73	0.82	27.89	28.32	0.42
LA public health	11.86	12.26	0.40	8.77	9.18	0.41	9.87	10.37	0.50
Number of CCGs and matched LAs	10	10	10	149	149	149	65	65	65

Abbreviations: GM, Greater Manchester; CCG, Clinical Commissioning Group; LA, local authority; SC, Synthetic control. LA data is mapped to CCG level on the basis of overlapping populations. ^a^London excluded from Rest of England. ^b^SC group is a weighted average of 65 CCGs from Rest of England (excluding London). Difference columns show absolute difference in mean between pre- and post- devolution periods.

 Compared with units from the rest of England, expenditure trends for the SC group followed a similar pattern to those of GM throughout the pre-devolution period (Supplementary file 2, Figures S1 and S2). Indeed, robustness tests identified no statistically significant differences in expenditure between GM and the SC group prior to devolution suggesting that the SC model was able to identify an appropriate control group for the DiD analysis (Supplementary file 1, Table S3).

###  Analysis

**Figure 1:**
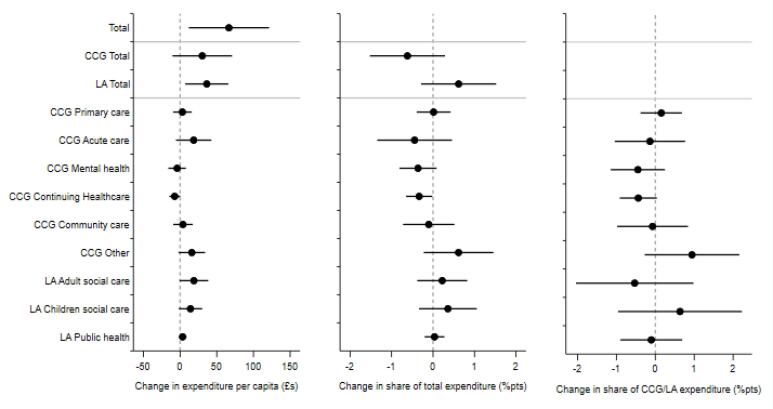
Coefficient Plots of the Estimated Average Effect of Greater Manchester Devolution on Expenditures. The figure presents estimates from the synthetic difference-in-differences model. Estimation sample (N = 525) includes 10 CCGs in GM and weighted combination of 65 CCGs from the rest of England over a 7-year period. Estimates show the average annual impact of devolution on expenditure in GM relative to the weighted synthetic control group throughout the four-year post-devolution period. Cluster-robust confidence intervals are presented at the 95 percent confidence level. Abbreviations: CCG, Clinical Commissioning group; LA, local authority; GM, Greater Manchester.

**Figure 2:**
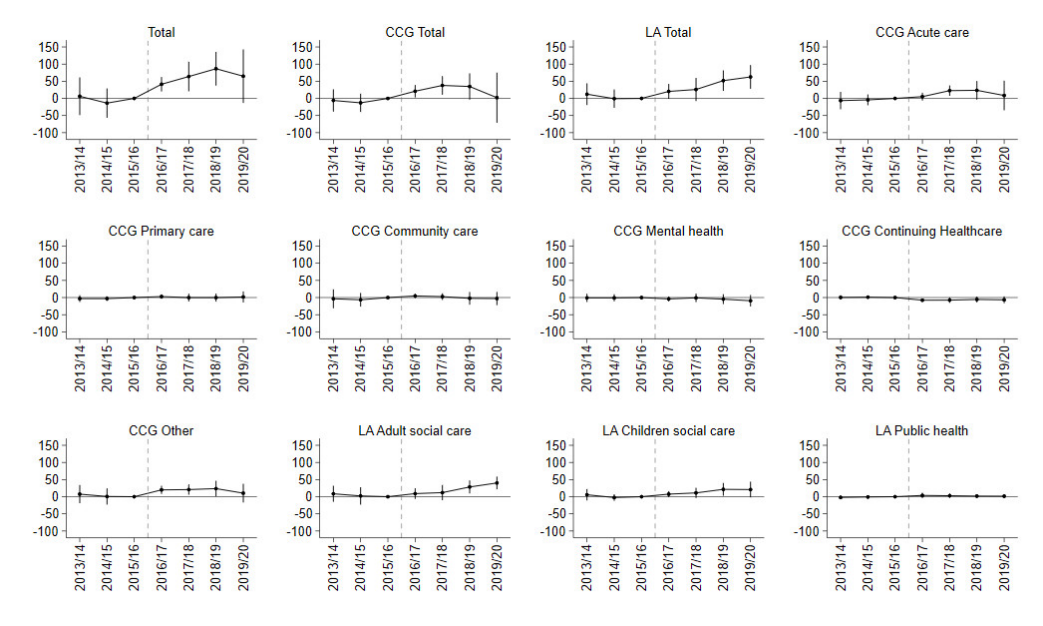
Event Study Plots of the Estimated Effect of Greater Manchester Devolution on Annual Per Capita Expenditures (£). The figure presents estimates from the synthetic difference-in-differences model. Estimation sample (N = 525) includes 10 CCGs in GM and weighted combination of 65 CCGs from the rest of England over a 7-year period. Estimates show the annual impact of devolution on expenditure in GM relative to the weighted synthetic control group throughout the four-year post-devolution period. Cluster-robust confidence intervals are presented at the 95 percent confidence level. Dashed vertical lines indicate when GM health and social care devolution deal came into force. Abbreviations: CCG, Clinical Commissioning group; LA, local authority; GM, Greater Manchester.

 Devolution was associated with a £66.58 per capita (standard error [SE] 27.47; *P* < .05) increase in total resources spent on health and care in GM on average in the post-intervention period, compared with the SC group from the rest of England (Figure 1, Table 2). The event study results show that this was driven primarily by increases in the first three years following devolution (Figure 2). Compared with the SC group, GM spent £41.30 (SE 10.59; *P* < .001) more on health and care in the first year post-devolution, £63.80 (SE 21.67; *P* < .01) more in the second year and £86.52 (SE 24.69; *P* < .001) more in the third year (Supplementary file 2, Table S4).

**Table 2:** Estimated Average Effects of Greater Manchester Devolution on Expenditures

	**Per Capita Expenditures (£)**		**Share of Total Expenditure (%pts)**		**Share of CCG/LA Expenditure (%pts)**	
Whole system						
Total	66.58*	(27.47)				
CCG						
Total	30.18	(20.37)	-0.62	(0.45)		
Primary care	3.17	(6.36)	0.01	(0.21)	0.15	(0.27)
Acute	18.52	(12.13)	-0.45	(0.45)	-0.14	(0.45)
Mental health	-4.08	(6.05)	-0.36	(0.23)	-0.45	(0.35)
Continuing healthcare	-7.32	(3.76)	-0.34*	(0.16)	-0.44	(0.24)
Community care	3.92	(6.68)	-0.10	(0.31)	-0.07	(0.46)
Other	15.97	(9.15)	0.62	(0.42)	0.94	(0.61)
LA						
Total	36.39*	(14.76)	0.62	(0.45)		
Adult social care	18.80	(9.83)	0.22	(0.30)	-0.53	(0.76)
Children’s social care	14.15	(8.04)	0.36	(0.35)	0.63	(0.80)
Public health	3.44	(2.70)	0.04	(0.12)	-0.10	(0.40)

Abbreviations: CCG, Clinical Commissioning Group; LA, local authority. Table 2 presents estimates from the synthetic difference-in-differences model. Estimation sample (n = 525) includes 10 CCGs in GM and weighted combination of 65 CCGs from the rest of England over a 7-year period. Estimates show the average annual impact of devolution on expenditure in GM relative to the weighted synthetic control group throughout the four-year post-devolution period. Cluster-robust standard errors at the CCG level are included in parentheses. *P < .05.

 The increase in expenditure was primarily from Local Authorities. The average effect of devolution on Local Authority per-capita expenditure on health and care was £36.39 (SE 14.76; *P* < .05). This was driven by the third- and fourth-years post-devolution, when GM Local Authorities spent an additional £51.79 (SE 15.01; *P* < .001) and £62.66 (SE 17.37; *P* < .001) respectively on health and care, compared with the SC group. By the fourth year post-devolution, the share of total health and care expenditure financed via Local Authorities had increased by 1.70 percentage points (SE 0.69; *P* < .05, Supplementary file 2, Table S5) more for GM.

 The average effect of devolution on adult social care expenditure in the post-devolution period (£18.80 per capita (SE 9.83) increase) was positive, but not statistically significant at the 5% level (Figure 1, Table 2). However, GM Local Authorities did spend more than the SC group on adult social care in the third (£28.42 [SE 9.53]; *P* < .01) and fourth (£40.15 [SE 9.36]; *P* < .001) years post-devolution (Supplementary file 2, Table S4). In the fourth-year post-devolution, the percentage of total health and care expenditure on adult social care increased by 1.16 percentage points (SE 0.36; *P* < .01; Supplementary file 2, Table S5). The average effect on expenditure on children’s social care was positive, with a £14.15 per capita increase (SE 8.04), although not statistically significant at the 5% level, and was higher in the third-year post-devolution (£21.51 [SE 9.39]; *P* < .05). There was a one percentage point decrease in the percentage of Local Authority health and care expenditure that was on public health (SE 0.45; *P* < .05) in the fourth-year post-devolution (Supplementary file 2, Table S6).

 GM CCGs’ total expenditure per capita increased by £20.95 (SE 9.12; *P* < .05) more than the SC group in the first-year post-devolution and £37.80 (SE 13.64; *P* < .01) in the second year (Figure 2; Supplementary file 2, Table S4). The average effect was £30.18 (SE 20.37), but this was not statistically significant at the 5% level. The effect on the share of total expenditure financed by CCGs mirrored that for Local Authorities; by the fourth-year post-devolution the percentage of total health and care expenditure financed by CCGs had reduced by 1.70 percentage points (SE 0.69; *P* < .05) more in GM compared with the SC group (Supplementary file 2, Table S5).

**Figure 3:**
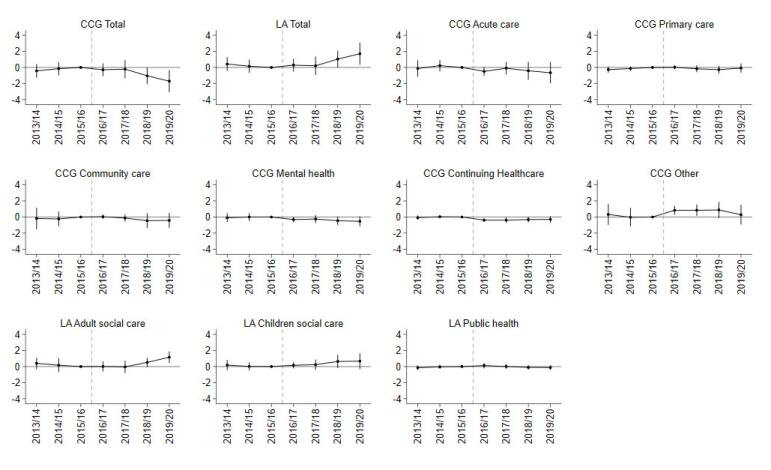
Event Study Plots of the Estimated Effect of Greater Manchester Devolution on Share of Total Health and Care Expenditure (%pts). The figure presents estimates from the synthetic difference-in-differences model. Estimation sample (N = 525) includes 10 CCGs in GM and weighted combination of 65 CCGs from the rest of England over a 7-year period. Estimates show the annual impact of devolution on expenditure in GM relative to the weighted synthetic control group throughout the four-year post-devolution period. Cluster-robust confidence intervals are presented at the 95 percent confidence level. Dashed vertical lines indicate when GM health and social care devolution deal came into force. Abbreviations: CCG, Clinical Commissioning group; LA, local authority; GM, Greater Manchester.

**Figure 4:**
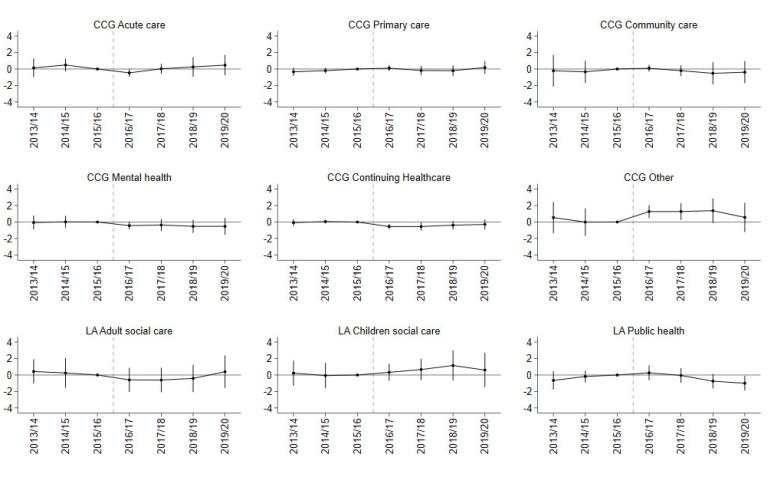
Event Study Plots of the Estimated Effect of Greater Manchester Devolution on Share of Total CCG/LA Expenditure (%pts). The figure presents estimates from the synthetic difference-in-differences model. Estimation sample (N = 525) includes 10 CCGs in GM and weighted combination of 65 CCGs from the rest of England over a 7-year period. Estimates show the annual impact of devolution on expenditure in GM relative to the weighted synthetic control group throughout the four-year post-devolution period. Cluster-robust confidence intervals are presented at the 95 percent confidence level. Dashed vertical lines indicate when GM health and social care devolution deal came into force. Abbreviations: CCG, Clinical Commissioning group; LA, local authority; GM, Greater Manchester.

 For primary care, community healthcare, and mental health services there was a small and not statistically significant change in per capita expenditure or the percentage share of total/CCG expenditure in GM relative to the SC group in any of the post-devolution years (Figures 2-4; Supplementary file 2, Tables S4-S6).

 GM spending on acute healthcare increased by £22.67 (SE7.59; *P* < .01) more in the second post-devolution year (Figure 2; Supplementary file 2, Table S4). The average effect was £18.52 (SE 12.13) but this was not statistically significant at the 5% level.

 CCG expenditure on the “other” category increased by £19.94 (SE 5.94; *P* < .01) more in the first year post-devolution, £20.83 (SE 7.58; *P* < .01) in the second year, and £23.69 (SE 11.45; *P* < .05) in the third year. The average effect was £15.97 per capita (SE 9.15), but it was not statistically significant at the 5% level. Expenditure on the “other” category also increased as a share of total expenditure by 0.84 percentage points (SE 0.28; *P* < .01) in the first year and 0.84 percentage points (SE 0.36; *P* < .05) in the second year (Figure 3; Supplementary file 2, Table S5) following devolution. Spending on the “other” category also increased as a share of CCG expenditure by 1.27 percentage points (SE 0.39; *P* < .01) in the third year and 1.27 percentage points (SE 0.51; *P* < .05), in the fourth year (Figure 4; Supplementary file 2, Table S6).

 CCG expenditure on Continuing Healthcare as a share of total expenditure decreased by 0.34 percentage points (SE 0.16; *P* < .05) on average for GM in the post period. This mainly occurred in the first (-0.39 [SE 0.10]; *P* < .001) and second years (-0.40 [SE 0.17]; *P* < .05) post-devolution. Expenditure on this category also decreased as a share of CCG expenditure by 0.44 percentage points (SE 0.24), more than in the control group, mainly in the first (-0.55, SE 0.15; *P* < .001) and second years (-0.55, SE 0.26; *P* < .05) post-devolution. In per capita terms, this difference corresponded to a £7.70 (SE 2.19; *P* < .001) reduction in spending on Continuing Healthcare in the first year following devolution (Supplementary file 2, Table S4).

###  Sensitivity Analysis

 When we tested whether the increase in total spending was driven by an increase in resources centrally allocated, we found no effect of devolution on core CCG allocations (Supplementary file 3, Table S7).

 Limiting the pool of control CCGs to those that received delegated co-commissioning responsibilities for primary care in the same years as GM CCGs reduced the number of CCGs in the control group to 42. Consistent with results from the main analysis, we also found no effect of devolution on this alternative definition of primary care expenditure (Supplementary file 3, Table S8).

 Moreover, the results were very similar to the main analysis when we excluded neighbouring regions from the pool of control units, indicating that results were unaffected by potential spillover effects from/on neighbouring regions (Supplementary file 3, Table S9).

## Discussion

 We used a synthetic DiD approach to estimate the impact of devolution on public expenditure on health and care in GM relative to comparable areas in the rest of England. We found that following devolution total expenditure on health and care increased more in GM compared to the SC group. The increase was distributed across different services. The share of expenditure financed by Local Authorities increased in GM, driven by higher expenditure on adult and children’s social care in the third- and fourth-years following devolution. We found a short-term increase in CCG spending on acute healthcare and “other” unclassified items and a short-term decrease in spending on Continuing Healthcare. The results were robust to sensitivity analyses.

 The increase in total expenditure was likely financed using mainly transformation funding from the devolution deal, driving observed improvements in health and health services.^17,20,34^ However, the 2019 end-of-year report for one Local Authority suggests that its social care “overspend” was partly funded by an underspend in non-health/care service lines,^48^ suggesting that spending on social care was also supplemented in other ways.

 There is no specific definition of CCG “other” miscellaneous spending. However, the concentration in the early post-devolution years suggests that it could have included health system transformation not attributable to specific service categories, such as costs of establishing data systems to enable integrated care.

 CCG expenditure on Continuing Healthcare reduced in the first year following devolution, both in per capita terms and proportionally. There was also limited evidence of changes in the resources that were allocated towards preventive services, despite increasing them being a strategic objective in GM.^16,21^ This may suggest that GM was unable to flexibly reallocate resources within the system, perhaps a reflection of still being subject to the same national rules, targets, and mechanisms for budget-pooling as elsewhere, or other regions pursuing similar agendas. The need to address existing pressures on services, particularly acute hospital services, may have also hindered GM shifting resources from those services to re-invest in others, without a sustained increase in available resources. However, increases in preventive spending could be captured in other categories, such as “miscellaneous” expenditure, making it harder to identify.

 The findings of increased expenditure on social care, acute services, and share of health and care resources financed by Local Authorities following GM devolution are in line with previous studies.^21^ However, we found no evidence of changes in public health and community healthcare expenditure, suggesting that previously cited reductions in these areas were not unique to GM. The finding of increased expenditure by Local Authorities on social care in the third- and fourth-year post-devolution may be related to the reduction in unpaid care found by a previous evaluation.^17^

###  Strengths

 We add to the limited evidence on the effects of devolution on health system financing. Our approach captures the objectives of the reform by examining the whole system of health and care expenditures.^16^ The synthetic DiD approach also allowed us to investigate both average and dynamic effects following devolution, comparing GM to a consistent control group with similar historic expenditure and population composition patterns. All models performed well, with tests supporting the assumption of parallel trends in the pre-intervention periods.

 We find that changes in expenditure following devolution were limited, and short-term, demonstrating the benefit of analysing changes in funding flows and considering dynamic effects. The changes were also concentrated in specific types of services, which demonstrates the importance of analysing expenditure on specific services when data availability permits it, rather than total expenditure only. Few existing studies have analysed service specific expenditure following devolution/decentralisation. The results of the present study and a similar study of decentralisation in Indonesia^14^ demonstrate that analyses limited to total expenditure can mask vastly different impacts on different services, which is of utmost relevance for understanding the overall health system impact of devolution policies.

###  Limitations

 The findings of this study should be considered in the context of its limitations. First, we were only able to consider expenditure for 2013/2014 onwards due to the establishment of CCGs in 2013. For this reason, we could include only three pre-devolution years in the synthetic DiD estimation which is fewer than optimal. Nonetheless, in line with previous studies we constructed the counterfactual using data on multiple measures of expenditure,^17,20^ thus reducing the possibility that the identified control group was matched on the noise of a single outcome.

 As with all policy evaluations, differences between GM and the SC group could have been driven by region-specific shocks that were unrelated to devolution, or unobserved time-varying factors related to underlying differences in the population of each region (eg, health behaviours). However, we are not aware of any other local interventions that would have affected GM differently from the rest of England. Tests for parallel trends showed that GM and the SC group followed similar trends across all expenditure measures throughout the pre-devolution period, lending weight to the assumptions of the model.

 The sample size of the study was necessarily limited by the number of CCG units in England. The relatively small number of units resulted in large standard errors for some estimated effects. Our analysis may have been underpowered to detect an effect for some outcomes.

 In addition, we used cluster-robust standard errors for inference which may have understated uncertainty given the small number of treated clusters and the region-level assignment of devolution.^49^ Caution should therefore be applied when interpreting the statistical significance of these findings, particularly for estimates with 95 percent confidence intervals close to zero. This mainly includes the estimates of average effects for the post-devolution period. Nonetheless, most estimates obtained from the event study estimation remained statistically significant when testing the statistical significance of the findings at the 99% confidence level, lending confidence to these results.

 CCGs and Local Authorities in GM may have classified expenditures differently to comparator organisations. To reduce the sensitivity of our analysis to minor differences in expenditure classification practices, we focussed only on major categories of expenditure likely to be broadly similar.

###  Policy Implications and Future Research

 There are some parallels between GM under devolution and Integrated Care Systems (ICSs), introduced in England in 2022, bringing together NHS organisations, local authorities, and voluntary and community partners to improve population health and reduce inequalities. The GM partnership was often seen as a ‘prototype’ of ICSs.^18^ GM and ICSs both have a major focus on prevention and service integration, and face similar challenges relating to initial integration costs (eg, costs of new digital infrastructure). However, there are several reasons that our estimated changes in expenditure in GM post-devolution are unlikely to be replicated by ICSs. First, ICSs are likely to face more difficult trade-offs than GM if they do not receive a similar level of transformation funding. It may be more challenging to redirect NHS and Local Authorities to support expenditure in one area without reductions elsewhere. In contrast, GM only reduced expenditure by a small amount in one service area in the short-term. Second, the changes in funding flows we observe in GM are suggestive of a relatively equal partnership between NHS organisations and local authorities, whereas early research on ICSs suggests unclear mechanisms for partnership and a potentially dominant role of NHS organisations.^50^ Integration may also be more challenging if local organisations do not have a strong history of collaboration as is the case in GM. GM organisations involved in the devolution agreement had a history of collaboration before the devolution deal, which may also affect generalisability to other contexts where this is not the case. The specific findings of this study may only be generalisable to similar contexts with comparable devolution policies.

 The effects we observe for GM may be attributable to some extent to devolution-linked transformation funding. However, that was not sufficient to substantially shift resources towards preventive services in line with GM strategic objectives.^16^ Additional resources and sufficient powers may be required to enable local health systems to shift patterns of spending. This is an important consideration for any country considering similar policies.

 More generally the results highlight the importance of analysing changes in funding flows across a whole health system under resource scarcity, where increases in expenditure in one area can adversely affect another. Future work should investigate the extent to which similar effects are observed in other settings following health and care devolution.

## Ethical issues

 This study used secondary data accessed at area-level. As no individual-level or identifiable data were used, ethical approval was not required.

## Conflicts of interest

 All authors had financial support from the National Institute for Health and Care Research (NIHR) Applied Research Collaboration for Greater Manchester for the submitted work; LA holds an honorary position with NHS England; no other relationships or activities that could appear to have influenced the submitted work.

## Supplementary files


Supplementary file 1 contains Tables S1-S3.

Supplementary file 2 contains Tables S4-S6 and Figures S1-S2.

Supplementary file 3 contains Tables S7-S9.
