# Supplementary material for: The Impact of Devolution on Local Health System Financing: A Synthetic Difference-in-Differences Study of Greater Manchester, England
Source: Int J Health Policy Manag. 2025 Sep 20;14:8689. doi: 10.34172/ijhpm.8689 (PMC12595567; doi:10.34172/ijhpm.8689)
Supplement: Supplementary file 1 — contains Tables S1-S3. [file ijhpm-14-8689-s001.pdf]

Article title: The Impact of Devolution on Local Health System Financing: A Synthetic Difference-in-Differences Study of Greater Manchester, England

Journal name: International Journal of Health Policy and Management (IJHPM)

Authors' information: Charlie Moss*, Philip Britteon, Yiu-Shing Lau, Laura Anselmi

Health Organisation, Policy and Economics (HOPE), Centre for Primary Care and Health Services Research, The University of Manchester, Manchester, UK

***Correspondence to:** Charlie Moss; Email: charlie.moss@manchester.ac.uk

Citation: Moss C, Britteon P, Lau YS, Anselmi L. The impact of devolution on local health system financing: a synthetic difference-in-differences study of Greater Manchester, England. Int J Health Policy Manag. 2025;14:8689. doi:10.34172/ijhpm.8689

Supplementary file 1

Supplementary file 1 – Synthetic differences-in-differences — Page

Table S1: Clinical Commissioning Groups included in synthetic control group and weights 2
Table S2: Predictor balance 5
Table S3: Tests of parallel trends 6

Table S1: Clinical Commissioning Groups included in synthetic control group and weights

CCG code	CCG name	W1	W2	W3	W4	W5	W6	W7	W8	W9	W10	GM weight
05Y	NHS Walsall CCG	0	0	0	0	0.343	0.081	0.26	0	0	0.196	**0.0999**
02W	NHS Bradford City CCG	0.035	0.107	0.242	0.034	0.253	0	0.093	0	0.005	0.074	**0.0789**
05L	NHS Sandwell and West Birmingham CCG	0	0	0	0	0	0	0.003	0	0	0.311	**0.0597**
04C	NHS Leicester City CCG	0	0.037	0	0	0	0	0	0	0	0.237	**0.0477**
09P	NHS Hastings and Rother CCG	0	0.053	0.425	0	0	0	0	0	0	0	**0.0387**
04K	NHS Nottingham City CCG	0	0	0.039	0.33	0	0	0	0	0	0	**0.0287**
04N	NHS Rushcliffe CCG	0.071	0.32	0	0	0	0	0	0	0	0	**0.0286**
10V	NHS South Eastern Hampshire CCG	0	0	0	0	0	0.27	0	0	0	0	**0.0278**
12D	NHS Swindon CCG	0.101	0	0	0	0.052	0	0.05	0	0.059	0.003	**0.0267**
12F	NHS Wirral CCG	0	0	0	0	0	0	0.278	0	0	0	**0.0254**
01A	NHS East Lancashire CCG	0	0	0	0	0	0.142	0	0.125	0	0	**0.0250**
00N	NHS South Tyneside CCG	0	0.109	0	0.015	0	0	0	0.031	0.119	0	**0.0248**
05A	NHS Coventry and Rugby CCG	0	0	0	0	0	0	0	0	0.211	0	**0.0243**
03J	NHS North Kirklees CCG	0.237	0	0	0	0	0	0	0	0	0	**0.0239**
02P	NHS Barnsley CCG	0	0.114	0.046	0.093	0.052	0	0	0	0	0	**0.0232**
99C	NHS North Tyneside CCG	0	0	0	0	0	0	0	0.271	0	0	**0.0226**
06P	NHS Luton CCG	0	0	0	0	0	0.021	0	0.037	0.002	0.084	**0.0215**
09C	NHS Ashford CCG	0	0	0	0.225	0	0	0	0	0	0	**0.0174**
06A	NHS Wolverhampton CCG	0	0.033	0	0	0.11	0.042	0	0	0	0	**0.0163**
10C	NHS Surrey Heath CCG	0.16	0	0	0	0	0	0	0	0	0	**0.0161**
09D	NHS Brighton and Hove CCG	0	0	0	0	0	0	0.004	0	0	0.081	**0.0158**
02N	NHS Airedale, Wharfedale and Craven CCG	0	0	0	0.204	0	0	0	0	0	0	**0.0157**
01R	NHS South Cheshire CCG	0	0	0	0	0	0.027	0.113	0.031	0	0	**0.0157**

05X	NHS Telford and Wrekin CCG	0.148	0	0	0	0	0	0	0	0	0	**0.0149**
02X	NHS Doncaster CCG	0	0	0.127	0	0	0.033	0	0	0	0	**0.0139**
15F	NHS Leeds CCG	0	0	0	0	0	0.047	0	0.107	0	0	**0.0138**
04L	NHS Nottingham North and East CCG	0	0	0.066	0.017	0	0.046	0	0	0	0	**0.0115**
03H	NHS North East Lincolnshire CCG	0	0	0	0	0	0	0	0.028	0.078	0	**0.0113**
00C	NHS Darlington CCG	0	0	0	0	0	0	0.12	0	0	0	**0.0109**
10X	NHS Southampton CCG	0	0	0	0	0	0	0	0	0.071	0.014	**0.0109**
15C	NHS Bristol, North Somerset and South Gloucestershire CCG	0	0	0	0	0	0	0	0.129	0	0	**0.0107**
10L	NHS Isle of Wight CCG	0	0	0	0	0	0.101	0	0	0	0	**0.0104**
99G	NHS Southend CCG	0	0	0	0	0	0	0	0.124	0	0	**0.0103**
05F	NHS Herefordshire CCG	0	0.077	0	0	0	0	0	0	0.041	0	**0.0099**
05C	NHS Dudley CCG	0.089	0	0	0	0.009	0	0	0	0	0	**0.0098**
06D	NHS Wyre Forest CCG	0	0	0.017	0.07	0	0	0.008	0	0.012	0	**0.0089**
05J	NHS Redditch and Bromsgrove CCG	0	0	0	0	0	0	0	0	0.072	0	**0.0083**
05G	NHS North Staffordshire CCG	0	0	0	0	0	0.08	0	0	0	0	**0.0082**
03T	NHS Lincolnshire East CCG	0	0	0	0	0	0	0	0	0.07	0	**0.0081**
09E	NHS Canterbury and Coastal CCG	0	0	0	0	0	0.078	0	0	0	0	**0.0080**
01T	NHS South Sefton CCG	0	0.076	0	0	0	0.027	0	0	0	0	**0.0079**
02D	NHS Vale Royal CCG	0	0	0	0.012	0.073	0	0	0	0	0	**0.0074**
15D	NHS East Berkshire CCG	0.072	0	0	0	0	0	0	0	0	0	**0.0073**
11M	NHS Gloucestershire CCG	0	0	0	0	0	0	0.036	0.043	0	0	**0.0069**
05N	NHS Shropshire CCG	0.065	0	0	0	0	0	0	0	0	0	**0.0066**
04H	NHS Newark & Sherwood CCG	0	0	0	0	0	0	0	0	0.051	0	**0.0059**
09N	NHS Guildford and Waverley CCG	0	0	0	0	0	0	0	0	0.05	0	**0.0058**
05H	NHS Warwickshire North CCG	0	0	0	0	0	0	0	0	0.048	0	**0.0055**
11E	NHS Bath and North East Somerset CCG	0	0	0	0	0	0	0	0	0.044	0	**0.0051**
01F	NHS Halton CCG	0	0.073	0	0	0	0	0	0	0	0	**0.0049**
03F	NHS Hull CCG	0	0	0	0	0	0	0	0.048	0	0	**0.0040**

05D	NHS East Staffordshire CCG	0	0	0	0	0.041	0	0	0	0	0	**0.0036**
03V	NHS Corby CCG	0	0	0.039	0	0	0	0	0	0	0	**0.0032**
05Q	NHS South East Staffordshire and Seisdon Peninsula CCG	0	0	0	0	0.035	0	0	0	0	0	**0.0031**
10K	NHS Fareham and Gosport CCG	0	0	0	0	0.032	0	0	0	0	0	**0.0028**
01V	NHS Southport and Formby CCG	0	0	0	0	0	0	0	0	0.024	0	**0.0028**
05V	NHS Stafford and Surrounds CCG	0	0	0	0	0	0	0	0	0.022	0	**0.0025**
09F	NHS Eastbourne, Hailsham and Seaford CCG	0.023	0	0	0	0	0	0	0	0	0	**0.0023**
01C	NHS Eastern Cheshire CCG	0	0	0	0	0	0	0.016	0.007	0	0	**0.0020**
01H	NHS Cumbria CCG	0	0	0	0	0	0	0.019	0	0	0	**0.0017**
04V	NHS West Leicestershire CCG	0	0	0	0	0	0	0	0.018	0	0	**0.0015**
06H	NHS Cambridgeshire and Peterborough CCG	0	0	0	0	0	0	0	0	0.012	0	**0.0014**
00R	NHS Blackpool CCG	0	0	0	0	0	0.006	0	0	0	0	**0.0006**
03D	NHS Hambleton, Richmondshire and Whitby CCG	0	0	0	0	0	0	0	0	0.005	0	**0.0006**
00M	NHS South Tees CCG	0	0	0	0	0	0	0	0	0.004	0	**0.0005**

Table S1 presents weights for 65 CCGs (out of 149 CCGs in the Rest of England) with a non-zero weighting in the main analysis. Weights derived using the synthetic control method. W1-W10 show weights for each of the individual CCGs in Greater Manchester; GM weight shows overall weights for GM that are used in the main analysis. Abbreviations: CCG, Clinical Commissioning Group; GM, Greater Manchester.

Table S2: Predictor balance

	Greater Manchester	Rest of England*	Synthetic control[†]
**Pre-devolution population composition**			
Female %	0	2.57	0.88
Female age	0	2.29	0.75
Male age	0	2.29	0.75
**2013 Per capita expenditure**			
CCG acute	-8.14	-4.02	-5.31
CCG Continuing Healthcare	-4.74	-5.55	-4.63
CCG community healthcare	-5.48	-7.5	-5.27
CCG mental health	-2.37	-0.9	-2.05
CCG other	0.02	-5.09	-4.77
CCG primary care	-7.78	-3.61	-6.75
LA adult social care	7.52	1.75	2.58
LA children's social care	-10.9	-18.03	-15.5
LA public health	-7.54	-5.19	-6.46
**2014 Per capita expenditure**			
CCG acute	-1.48	0.29	-0.75
CCG Continuing Healthcare	0.94	-0.29	0.33
CCG community healthcare	-4.02	-2.48	-0.9
CCG mental health	-0.17	-0.91	0.09
CCG other	-10.98	-5.71	-8.92
CCG primary care	-0.56	1.37	0.46
LA adult social care	-6.19	-3.62	-4.86
LA children's social care	6.38	8.54	9.86
LA public health	-1.57	-1.61	-1.74
**2015 Per capita expenditure**			
CCG acute	9.62	3.73	6.06
CCG Continuing Healthcare	3.8	5.84	4.29
CCG community healthcare	9.5	9.97	6.17
CCG mental health	2.55	1.81	1.96
CCG other	10.95	10.8	13.7
CCG primary care	8.34	2.24	6.29
LA adult social care	-1.33	1.87	2.28
LA children's social care	4.52	9.49	5.64
LA public health	9.11	6.8	8.2

Table S2 shows the mean value of predictor variables used to define the synthetic control group. LA data is mapped to CCG level on the basis of overlapping populations. *London excluded from Rest of England. [†]Synthetic control group is a weighted average of 65 CCGs from Rest of England (excluding London). Abbreviations: CCG, Clinical Commissioning Group; LA, local authority. LA data is mapped to CCG level on the basis of overlapping populations.

Table S3: Tests of parallel trends

Outcome	F test P-value	Linear trend p-value
Per capita expenditure		
Total	0.41	0.82
CCG total	0.26	0.71
CCG primary care	0.64	0.54
CCG acute	0.86	0.62
CCG mental health	0.99	0.88
CCG Continuing Healthcare	0.78	0.90
CCG community health services	0.67	0.80
CCG other	0.13	0.57
LA total	0.60	0.44
LA adult social care	0.64	0.47
LA child social care	0.66	0.50
LA public health	0.70	0.48
Shares of total health and care expenditure		
CCG total	0.60	0.33
CCG primary care	0.39	0.17
CCG acute	0.57	0.80
CCG mental health	0.82	0.74
CCG Continuing Healthcare	0.49	0.59
CCG community health services	0.83	0.79
CCG other	0.09	0.62
LA total	0.60	0.33
LA adult social care	0.45	0.27
LA child social care	0.85	0.57
LA public health	0.44	0.31
Shares of total CCG/LA expenditure		
CCG primary care	0.41	0.19
CCG acute	0.40	0.81
CCG mental health	0.93	0.89
CCG Continuing Healthcare	0.61	0.72
CCG community health services	0.79	0.83
CCG other	0.07	0.57
LA adult social care	0.84	0.56
LA child social care	0.94	0.76
LA public health	0.44	0.25

Table S3 presents results of an F test of the joint significance of differences in expenditure in each period, and a test of the differences between linear trends in expenditure throughout the pre-devolution period (2013/14 to 2019/20). Sample (N=225) includes 10 CCGs in GM and weighted combination of 65 CCGs from the rest of England over a 3-year period. Abbreviations: CCG, Clinical Commissioning group; LA, Local Authority.
