# Supplementary material for: The Impact of Devolution on Local Health System Financing: A Synthetic Difference-in-Differences Study of Greater Manchester, England
Source: Int J Health Policy Manag. 2025 Sep 20;14:8689. doi: 10.34172/ijhpm.8689 (PMC12595567; doi:10.34172/ijhpm.8689)
Supplement: Supplementary file 2 — contains Tables S4-S6 and Figures S1-S2. [file ijhpm-14-8689-s002.pdf]

Article title: The Impact of Devolution on Local Health System Financing: A Synthetic Difference-in-Differences Study of Greater Manchester, England

Journal name: International Journal of Health Policy and Management (IJHPM)

Authors' information: Charlie Moss*, Philip Britteon, Yiu-Shing Lau, Laura Anselmi

Health Organisation, Policy and Economics (HOPE), Centre for Primary Care and Health Services Research, The University of Manchester, Manchester, UK

***Correspondence to:** Charlie Moss; Email: charlie.moss@manchester.ac.uk

Citation: Moss C, Britteon P, Lau YS, Anselmi L. The impact of devolution on local health system financing: a synthetic difference-in-differences study of Greater Manchester, England. Int J Health Policy Manag. 2025;14:8689. doi:10.34172/ijhpm.8689

Supplementary file 2

Supplementary file 2 – Results | Page

Supplementary file 2 – Results	Page
Figure S1: Trends over time, per capita expenditures (£)	2
Figure S2: Trends over time, share of total expenditure (%)	3
Table S4: Estimated effects of devolution on annual per capita expenditures (£): event study results	4
Table S5: Estimated effects of devolution on share of total health and care expenditure (%pts): event study results	5
Table S6: Estimated effects of devolution on share of Clinical Commissioning Group/local authority health and care expenditure (%pts): event study results	6

Figure S1: Trends over time, per capita expenditures (£)

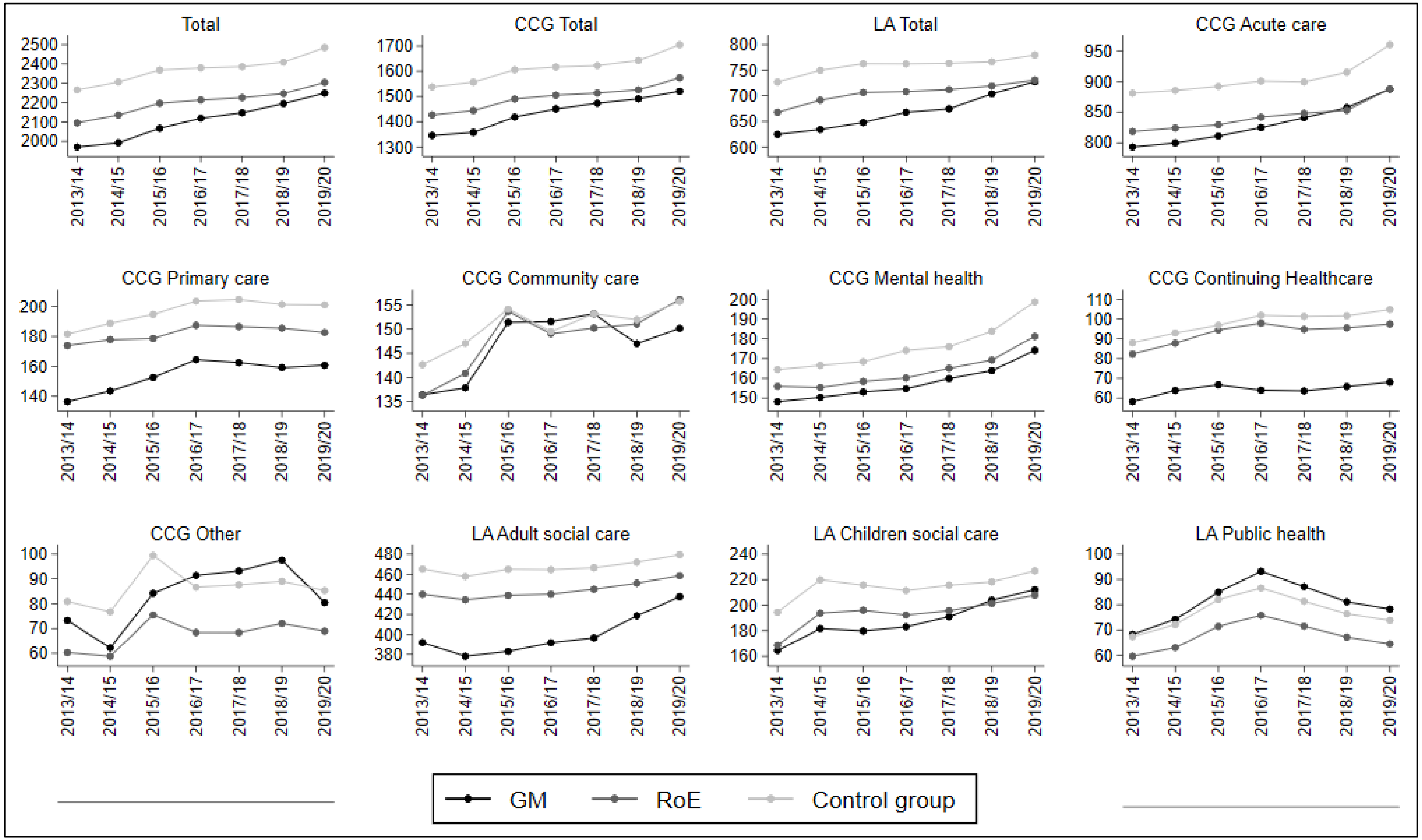

Figure S1 shows mean per capita expenditures over a seven-year period. Abbreviations: CCG, Clinical Commissioning group; LA, local authority; RoE, Rest of England. LA data is mapped to CCG level on the basis of overlapping populations. London excluded from RoE. GM includes 10 CCGs. RoE includes 149 CCGs. Synthetic control group is a weighted average of 65 CCGs from Rest of England (excluding London).

Figure S2: Trends over time, share of total expenditure (%)

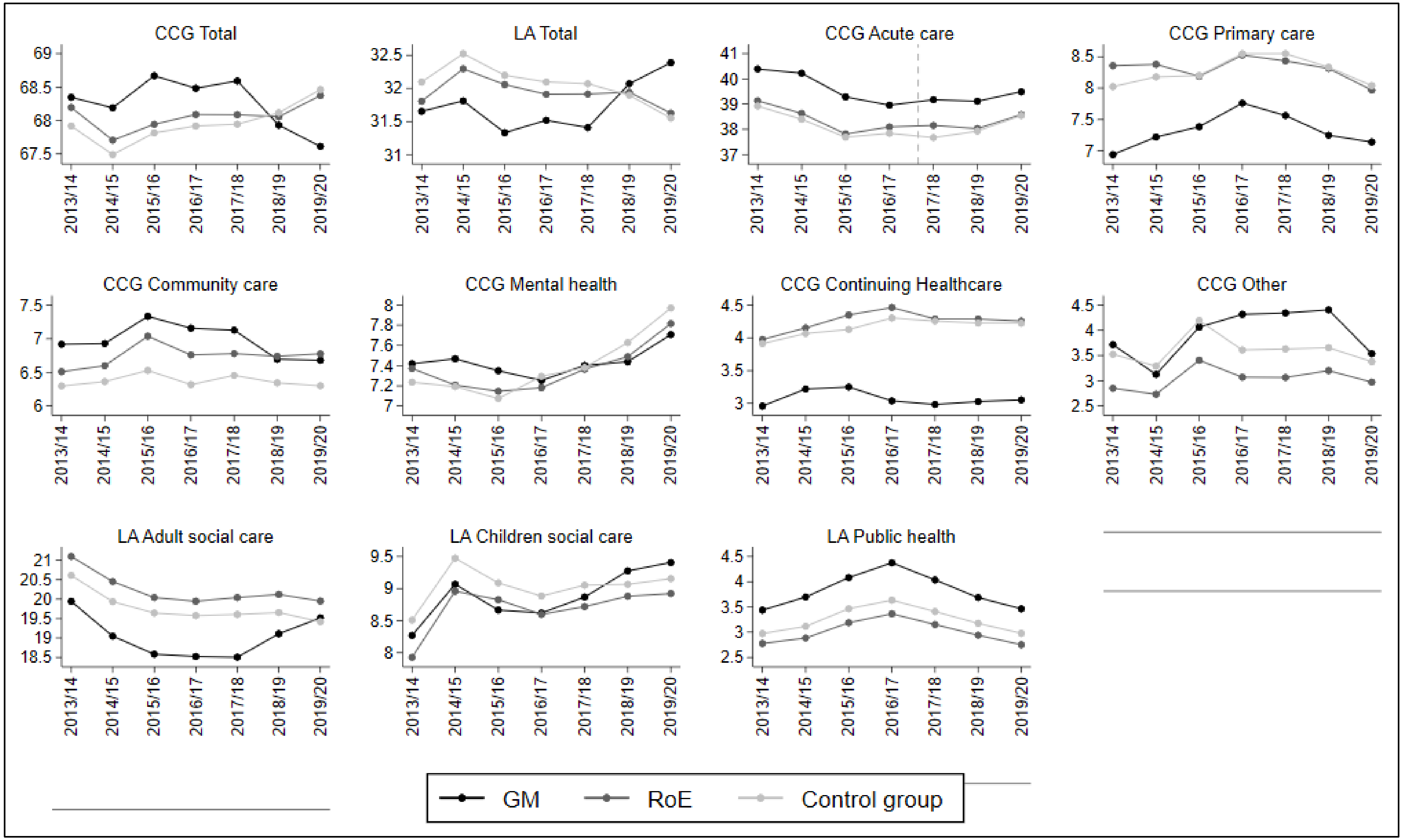

Figure S2 shows mean share of total health and care expenditure absorbed by different services over a seven-year period. Abbreviations: CCG, Clinical Commissioning group; LA, local authority; RoE, Rest of England. LA data is mapped to CCG level on the basis of overlapping populations. London excluded from RoE. GM includes 10 CCGs. RoE includes 149 CCGs. Synthetic control group is a weighted average of 65 CCGs from Rest of England (excluding London).

Table S4: Estimated effects of Greater Manchester devolution on annual per capita expenditures (£): event study results

	Clinical Commissioning Group							Local authority				Total
	**Primary care**	**Acute**	**Mental health**	**Continuing Healthcare**	**Community health services**	**Other**	**Total**	**Adult social care**	**Children's social care**	**Public health**	**Total**	
**2013**	-3.08	-6.39	-0.91	0.38	-3.54	7.54	-5.99	8.55	5.71	-2.00	12.26	6.27
	(4.98)	(12.72)	(6.01)	(3.11)	(13.85)	(13.34)	(16.32)	(11.74)	(8.36)	(2.80)	(15.91)	(27.59)
**2014**	-3.07	-4.28	-0.84	1.10	-6.45	0.69	-12.85	2.28	-2.37	-0.75	-0.85	-13.69
	(3.34)	(7.90)	(5.15)	(1.79)	(10.02)	(11.88)	(13.41)	(12.76)	(4.81)	(2.64)	(13.45)	(21.47)
**(Omitted)**												
**2016**	2.93	5.06	-4.02	-7.70***	4.74	19.94**	20.95*	9.06	7.54	3.75	20.35	41.30***
	(3.15)	(5.68)	(3.63)	(2.19)	(3.06)	(5.93)	(9.12)	(7.78)	(4.41)	(3.92)	(10.95)	(10.59)
**2017**	-0.12	22.67**	-0.78	-7.51	2.71	20.83**	37.80**	12.02	11.10	2.88	26.00	63.80**
	(5.69)	(7.59)	(6.20)	(3.97)	(4.91)	(7.58)	(13.64)	(11.16)	(7.48)	(3.26)	(16.94)	(21.67)
**2018**	-0.12	23.62	-4.64	-5.55	-2.27	23.69*	34.73	28.42**	21.51*	1.86	51.79***	86.52***
	(5.92)	(13.61)	(7.22)	(4.30)	(9.32)	(11.45)	(19.24)	(9.53)	(9.39)	(2.72)	(15.01)	(24.69)
**2019**	1.79	8.49	-9.21	-6.53	-2.83	10.41	2.12	40.15***	20.92	1.60	62.66***	64.78
	(8.06)	(21.67)	(8.39)	(4.96)	(9.70)	(13.66)	(36.69)	(9.36)	(11.52)	(2.85)	(17.37)	(39.20)

Table S4 presents estimates from the synthetic difference-in-differences model. Estimation sample (N=525) includes 10 CCGs in GM and weighted combination of 65 CCGs from the rest of England over a 7-year period. Estimates show the annual impact of devolution on expenditure in GM relative to the weighted synthetic control group throughout the four-year post-devolution period. Cluster-robust standard errors at the CCG level are included in parentheses. Abbreviations: CCG, Clinical Commissioning group; LA, local authority. *p<0.05, **p<0.01, ***p<0.001.

Table S5: Estimated effects of Greater Manchester devolution on share of total health and care expenditure (%pts): event study results

	Clinical Commissioning Group							Local authority			
	**Primary care**	**Acute**	**Mental health**	**Continuing Healthcare**	**Community health services**	**Other**	**Total**	**Adult social care**	**Children's social care**	**Public health**	**Total**
**2013**	-0.27	-0.13	-0.09	-0.07	-0.18	0.32	-0.42	0.39	0.18	-0.15	0.42
	(0.19)	(0.52)	(0.28)	(0.14)	(0.67)	(0.65)	(0.43)	(0.36)	(0.32)	(0.15)	(0.43)
**2014**	-0.14	0.23	0.00	0.03	-0.24	-0.03	-0.15	0.17	0.02	-0.04	0.15
	(0.13)	(0.36)	(0.25)	(0.08)	(0.46)	(0.57)	(0.41)	(0.43)	(0.26)	(0.12)	(0.41)
**(Omitted)**											
**2016**	0.03	-0.49	-0.32	-0.39***	0.04	0.84**	-0.29	0.01	0.16	0.12	0.29
	(0.13)	(0.28)	(0.17)	(0.10)	(0.13)	(0.28)	(0.40)	(0.32)	(0.19)	(0.17)	(0.40)
**2017**	-0.17	-0.10	-0.25	-0.40*	-0.13	0.84*	-0.20	-0.04	0.24	0.01	0.20
	(0.21)	(0.39)	(0.25)	(0.17)	(0.23)	(0.36)	(0.58)	(0.38)	(0.32)	(0.14)	(0.58)
**2018**	-0.27	-0.41	-0.46	-0.32	-0.45	0.88	-1.04	0.51	0.63	-0.11	1.04
	(0.24)	(0.56)	(0.27)	(0.17)	(0.47)	(0.51)	(0.52)	(0.29)	(0.41)	(0.14)	(0.52)
**2019**	-0.08	-0.65	-0.54	-0.29	-0.42	0.29	-1.70*	1.16**	0.67	-0.13	1.70*
	(0.29)	(0.66)	(0.31)	(0.20)	(0.46)	(0.61)	(0.69)	(0.36)	(0.50)	(0.15)	(0.69)

Table S5 presents estimates from the synthetic difference-in-differences model. Estimation sample (N=525) includes 10 CCGs in GM and weighted combination of 65 CCGs from the rest of England over a 7-year period. Estimates show the annual impact of devolution on expenditure in GM relative to the weighted synthetic control group throughout the four-year post-devolution period. Cluster-robust standard errors at the CCG level are included in parentheses. Abbreviations: CCG, Clinical Commissioning group; LA, local authority. *p<0.05, **p<0.01, ***p<0.001.

Table S6: Estimated effects of Greater Manchester devolution on share of Clinical Commissioning Group/local authority health and care expenditure (%pts): event study results

	Clinical Commissioning Group						Local authority		
	**Primary care**	**Acute**	**Mental health**	**Continuing Healthcare**	**Community health services**	**Other**	**Adult social care**	**Children's social care**	**Public health**
**2013**	-0.34	0.14	-0.06	-0.07	-0.21	0.54	0.43	0.23	-0.66
	(0.25)	(0.57)	(0.42)	(0.21)	(0.96)	(0.95)	(0.74)	(0.77)	(0.56)
**2014**	-0.20	0.49	0.02	0.05	-0.35	-0.01	0.25	-0.07	-0.18
	(0.17)	(0.39)	(0.37)	(0.11)	(0.68)	(0.83)	(0.91)	(0.78)	(0.36)
**(Omitted)**									
**2016**	0.10	-0.47	-0.44	-0.55***	0.09	1.27**	-0.59	0.33	0.27
	(0.18)	(0.24)	(0.24)	(0.15)	(0.21)	(0.39)	(0.73)	(0.52)	(0.46)
**2017**	-0.18	0.02	-0.36	-0.55*	-0.20	1.27*	-0.61	0.66	-0.05
	(0.28)	(0.31)	(0.36)	(0.26)	(0.33)	(0.51)	(0.75)	(0.66)	(0.44)
**2018**	-0.20	0.26	-0.53	-0.38	-0.52	1.37	-0.41	1.16	-0.75
	(0.33)	(0.60)	(0.40)	(0.25)	(0.68)	(0.75)	(0.83)	(0.92)	(0.44)
**2019**	0.18	0.47	-0.53	-0.29	-0.39	0.56	0.40	0.60	-1.00*
	(0.39)	(0.62)	(0.51)	(0.30)	(0.68)	(0.89)	(0.99)	(1.04)	(0.45)

Table S6 presents estimates from the synthetic difference-in-differences model. Estimation sample (N=525) includes 10 CCGs in GM and weighted combination of 65 CCGs from the rest of England over a 7-year period. Estimates show the annual impact of devolution on expenditure in GM relative to the weighted synthetic control group throughout the four-year post-devolution period. Cluster-robust standard errors at the CCG level are included in parentheses. Abbreviations: CCG, Clinical Commissioning group; LA, local authority. *p<0.05, **p<0.01, ***p<0.001.
