# Supplementary material for: The Impact of Devolution on Local Health System Financing: A Synthetic Difference-in-Differences Study of Greater Manchester, England
Source: Int J Health Policy Manag. 2025 Sep 20;14:8689. doi: 10.34172/ijhpm.8689 (PMC12595567; doi:10.34172/ijhpm.8689)
Supplement: Supplementary file 3 — contains Tables S7-S9. [file ijhpm-14-8689-s003.pdf]

Article title: The Impact of Devolution on Local Health System Financing: A Synthetic Difference-in-Differences Study of Greater Manchester, England

Journal name: International Journal of Health Policy and Management (IJHPM)

Authors' information: Charlie Moss*, Philip Britteon, Yiu-Shing Lau, Laura Anselmi

Health Organisation, Policy and Economics (HOPE), Centre for Primary Care and Health Services Research, The University of Manchester, Manchester, UK

***Correspondence to:** Charlie Moss; Email: charlie.moss@manchester.ac.uk

Citation: Moss C, Britteon P, Lau YS, Anselmi L. The impact of devolution on local health system financing: a synthetic difference-in-differences study of Greater Manchester, England. Int J Health Policy Manag. 2025;14:8689. doi:10.34172/ijhpm.8689

Supplementary file 3

**Supplementary file 3 – Results (sensitivity analyses)**	**Page**
Table S7: The estimated effect of devolution on Clinical Commissioning Group core allocations	2
Table S8: Estimated average effect of devolution on CCG primary care expenditure	3
Table S9: Estimated average effects of devolution on expenditures, excluding neighbouring regions	4

Table S7: Estimated effects of devolution on Clinical Commissioning Group core allocations

	**Core CCG allocations (£ per capita)**
**2013**	-8.81 (11.29)
**2014**	-9.25 (8.74)
**(Omitted)**	
**2016**	7.95 (7.52)
**2017**	10.91 (8.67)
**2018**	16.47 (21.53)
**2019**	-4.07 (31.61)
**Average effect**	13.83 (19.09)

Table S7 presents estimates from the synthetic difference-in-differences model. Estimation sample (N=525) includes 10 CCGs in GM and weighted combination of 65 CCGs from the rest of England over a 7-year period. Estimates show the annual impact of devolution on core CCG allocations in GM relative to the weighted synthetic control group throughout the four-year post-devolution period. Cluster-robust standard errors at the CCG level are included in parentheses. Abbreviations: CCG, Clinical Commissioning group; LA, local authority. *p<0.05.

Table S8: Estimated effects of devolution on CCG primary care expenditure

	**Per capita (£)**	**Share of total expenditure (%pts)**	**Share of CCG expenditure (%pts)**
**2013**	-0.68 (22.11)	-0.15 (0.82)	-0.06 (1.10)
**2014**	-0.95 (22.39)	-0.06 (0.84)	0.04 (1.13)
**(Omitted)**			
**2016**	-10.74 (20.44)	-0.07 (0.76)	-0.13 (0.99)
**2017**	-8.68 (24.57)	-0.23 (0.93)	-0.26 (1.20)
**2018**	-6.11 (25.32)	-0.32 (0.95)	-0.26 (1.24)
**2019**	-11.40 (23.35)	-0.46 (0.83)	-0.29 (1.06)
Average effect	-8.69 (9.74)	-0.20 (0.38)	-0.23 (0.46)

Table S8 presents estimates from the synthetic difference-in-differences model. Estimation sample (N=364) includes 10 CCGs in GM and weighted combination of 42 CCGs from the rest of England over a 7-year period. The control group is limited to CCGs that received delegated commissioning responsibilities for primary care in the same years as Greater Manchester CCGs (2015/16 and 2016/17). Estimates show the annual impact of devolution on CCG primary care expenditure in GM relative to the weighted synthetic control group throughout the four-year post-devolution period. Cluster-robust standard errors at the CCG level are included in parentheses. Abbreviations: CCG, Clinical Commissioning group; LA, local authority. *p<0.05.

.

Table S9: Estimated average effects of devolution on expenditures, excluding neighbouring regions

	Per capita expenditures (£)		Share of total expenditure (%pts)		Share of CCG/LA expenditure (%pts)	
**<u>Whole system</u>**						
Total	61.75*	(27.63)				
**<u>CCG</u>**						
Total	28.28	(21.14)	-0.55	(0.46)		
Primary care	3.42	(6.64)	0.05	(0.21)	0.19	(0.27)
Acute	15.44	(12.44)	-0.50	(0.45)	-0.28	(0.45)
Mental health	-4.61	(6.14)	-0.37	(0.23)	-0.46	(0.35)
Continuing Healthcare	-8.38	(3.86)	-0.37*	(0.16)	-0.49	(0.24)
Community care	5.06	(6.76)	-0.04	(0.31)	0.01	(0.46)
Other	17.36	(9.35)	0.68	(0.43)	1.04	(0.63)
**<u>LA</u>**						
Total	33.47*	(14.76)	0.55	(0.46)		
Adult social care	16.13	(9.87)	0.14	(0.31)	-0.65	(0.76)
Children's social care	13.85	(8.06)	0.36	(0.35)	0.70	(0.81)
Public health	3.49	(2.69)	0.05	(0.12)	-0.05	(0.40)

Table S9 presents estimates from the synthetic difference-in-differences model. Estimation sample (N=560) includes 10 CCGs in GM and weighted combination of 70 CCGs from the rest of England over a 7-year period. CCGs bordering GM are excluded from pool of potential control units. Estimates show the average annual impact of devolution on expenditure in GM relative to the weighted synthetic control group throughout the four-year post-devolution period. Cluster-robust standard errors at the CCG level are included in parentheses. Abbreviations: CCG, Clinical Commissioning group; LA, local authority. *p<0.05
